# Supplementary material for: Development of a yeast whole-cell biocatalyst for MHET conversion into terephthalic acid and ethylene glycol
Source: Microb Cell Fact. 2022 Dec 31;21:280. doi: 10.1186/s12934-022-02007-9 (PMC9805092; doi:10.1186/s12934-022-02007-9)
Supplement: Supplementary file 1 — Additional file 1: Figure S1. Mrh1 transmembrane topology prediction. Results obtained from the DeepTMHMM application (dtu.biolib.com/DeepTMHMM, accessed September 28, 2022) for the Mrh1 protein. Top panel: protein domain orientation relative to the inner and outer part of the cytoplasmic membrane. Bottom panel: probability associated with the inner and outer orientation for each protein domain. Figure S2. Para-nitrophenol extinction coefficient modeling. A. Extinction coefficient curve fitting. Discreet extinction coefficient data from Biggs 1954 [50] was used to model para-nitrophenol extinction coefficients between pH 6 and 10. B. Extinction coefficients for para-nitrophenol at the indicated pH used in this study based on modelling shown in A. Figure S3. Purified recombinant MHETase. Molecular weights of reference markers in kDa are indicated. [file 12934_2022_2007_MOESM1_ESM.pdf]

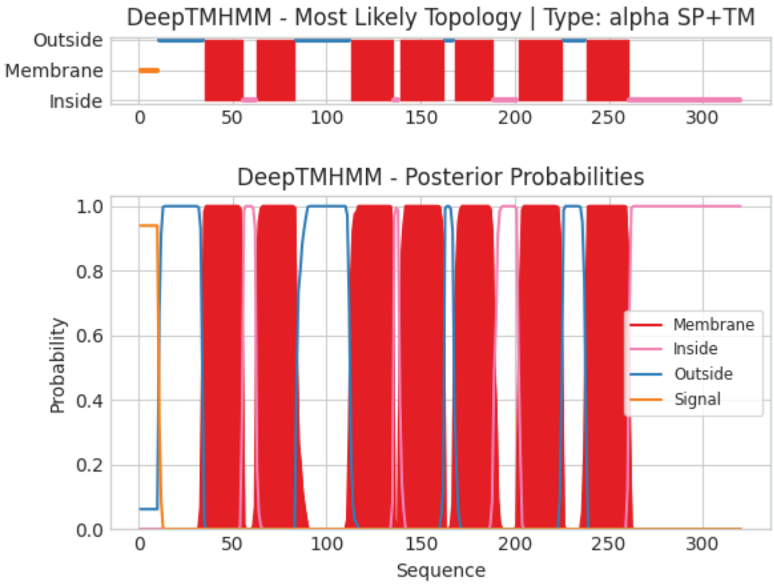

Figure S1. Mrh1 transmembrane topology prediction. Results obtained from the DeepTMHMM application (dtu.biolib.com/DeepTMHMM, accessed September 28, 2022) for the Mrh1 protein. Top panel: protein domain orientation relative to the inner and outer part of the cytoplasmic membrane. Bottom panel: probability associated with the inner and outer orientation for each protein domain.

A

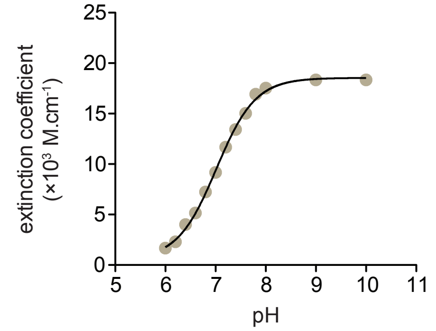

B

	ε pNP (M.cm^{-1})
pH = 7.5	14522
pH = 8.5	18156
pH = 9.5	18512
pH = 10.5	18534

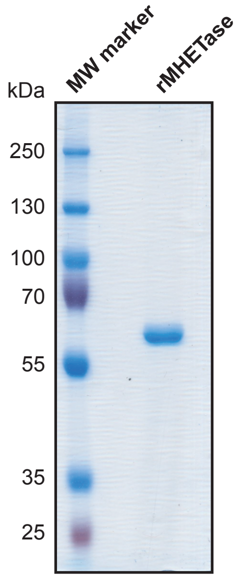

Figure S2. para-nitrophenol extinction coefficient modeling. A. Extinction coefficient curve fitting. Discreet extinction coefficient data from Biggs 1954 [50] was used to model para-nitrophenol extinction coefficients between pH 6 and 10. **B.** Extinction coefficients for para-nitrophenol at the indicated pH used in this study based on modelling shown in A.

Figure S3. Purified recombinant MHETase. Molecular weights of reference markers in kDa are indicated
