# Supplementary material for: Development of a yeast whole-cell biocatalyst for MHET conversion into terephthalic acid and ethylene glycol
Source: Microb Cell Fact. 2022 Dec 31;21:280. doi: 10.1186/s12934-022-02007-9 (PMC9805092; doi:10.1186/s12934-022-02007-9)
Supplement: Supplementary file 2 — Additional file 2: Table S1. Yeast strains used in this study. Table S2. DNA sequences of the MHETase surface display chimeras and display partners. [file 12934_2022_2007_MOESM2_ESM.pdf]

Table S1. Yeast strains used in this study.

strain ID	shorthand	genotype
DHY213	-	*MAT***a** *CAT5(91M) SAL1 MIP1(661T) HAP1 MKT1(30G) RME1(INS-308A) TAO3(1493Q) leu2Δ0 his3Δ1 ura3Δ0 met15Δ0*
RLKY218	-	*MAT***a** *CAT5(91M) SAL1 MIP1(661T) HAP1 MKT1(30G) RME1(INS-308A) TAO3(1493Q) leu2Δ0 his3Δ1 ura3Δ0 met15Δ0 leu2Δ::RNR2pr-tetR-NLS-tup1-7tet.1pr-tetR-NLS[LEU2]*
RLKY229	SED1	*RLKY218 can1Δ::WTC846pr-OST1-GFP-TEV-myc-SED1-tPRM9*
RLKY234	AGA2	*RLKY218 can1Δ::WTC846pr-OST1-GFP-TEV-myc-AGA2-tPRM9*
RLKY230	CCW12	*RLKY218 can1Δ::WTC846pr-OST1-GFP-TEV-myc-CCW12-tPRM9*
RLKY235	CIS3	*RLKY218 can1Δ::WTC846pr-OST1-GFP-TEV-myc-CIS3-tPRM9*
RLKY231	TIP1	*RLKY218 can1Δ::WTC846pr-OST1-GFP-TEV-myc-TIP1-tPRM9*
RLKY232	CWP2	*RLKY218 can1Δ::WTC846pr-OST1-GFP-TEV-myc-CWP2-tPRM9*
RLKY224	M-AGA2	*RLKY218 can1Δ::WTC846pr-OST1-MHETase-GFP-TEV-myc-AGA2-tPRM9*
RLKY225	M-CCW12	*RLKY218 can1Δ::WTC846pr-OST1-MHETase-GFP-TEV-myc-CCW12-tPRM9*
RLKY226	M-CIS3	*RLKY218 can1Δ::WTC846pr-OST1-MHETase-GFP-TEV-myc-CIS3-tPRM9*
RLKY227	M-TIP1	*RLKY218 can1Δ::WTC846pr-OST1-MHETase-GFP-TEV-myc-TIP1-tPRM9*
RLKY228	M-CWP2	*RLKY218 can1Δ::WTC846pr-OST1-MHETase-GFP-TEV-myc-CWP2-tPRM9*
RLKY244	M-SED1	*RLKY218 can1Δ::WTC846pr-OST1-MHETase-GFP-TEV-myc-SED1-tPRM9*
RLKY245	intra-M	*RLKY218 can1Δ::WTC846pr-MHETase-GFP-TEV-myc-tPRM9*
RLKY247	secretion-M	*RLKY218 can1Δ::WTC846pr-OST1-MHETase-GFP-TEV-myc-tPRM9*
GFP library strains		*MAT***a** *xxx::GFP[HIS3MX] leu2Δ0 his3Δ1 ura3Δ0 met15Δ0*

Table S2. DNA sequences of the MHETase surface display chimeras and display partners

MHETase chimera sequences:
acgccgccatccagtgtttaaacgaactagtgcggccgccagttcgagtttatcattatcaata
ctgccatttcaaagaatacgtaaataattaatagtagtgattttcctaactttatttagtcaaa
aaattagcctctatcattgatagagtgtctgggtgatctatcattgatagagcatccactaatt
ttaattctgctgtaacccgtacatgcccaaaatagggggcgggttacacagaatctatcattga
tagagtgtctgggtgatctatcattgatagagcatccactaaatataatggagctctatcattg
atagagcatccactaaaaaaaagaatcccagcaccaaaatattgttttcttcaccaaccatcag
ttcataggtccattctcttagcgcaactacagagaacaggggcacaaacaggcaaaaaacgggc
acaacctcaatggagtgatgcaacctgcctggagtaaatgatgacacaaggcaattgacccacg
catgtatctatctcattttcttacaccttctattaccttctgctctctctgatttggaaaaagc
tgaaaaaaaaggttgaaaccagttccctgaaattattcccctatctatcattgatagatataaa
tatctatcattgatagagtaattctgtaaatctatttcttaaacttcttaaattctactttat
agttagtcttttttttagttttaaaacaccaagaacttagtttcgaataaacacacataaaca
aaATGCGTCAAGTCTGGTTTTCTTGGATTGTTGGATTGTTTTTATGCTTTTTTAATGTTTCATC
GGCAGGTGGCGGAAGCACACCCCTACCCCTACCACAACAACAACCGCCCCAGCAGGAGCCCCCT
CCACCACCGGTACCACTAGCTTCAAGGGCGGCATGTGAAGCTTTGAAAGATGGCAATGGAGACA
TGGTTTGGCCGAATGCTGCTACTGTGGTTGAGGTGGCTGCATGGCGTGACGCCGCGCCCGCTAC
TGCAAGTGCGGCGGCTCTGCCTGAGCATTGTGAAGTATCAGGTGCTATAGCGAAACGTACTGGA
ATCGACGGATATCCCTATGAAATTAAGTTTCGTCTACGTATGCCAGCTGAATGGAATGGCCGTT
TCTTTATGGAAGGTGGGAGTGGTACGAATGGCAGCCTAAGTGCGGCAACCGGAAGCATTGGCGG
AGGGCAGATTGCCTCCGCTCTATCCAGGAACTTTGCAACGATAGCTACGGACGGTGGCCATGAT
AACGCTGTAAATGACAATCCCGACGCCTTGGGCACAGTAGCATTCGGATTAGATCCCCAGGCAA
GACTTGATATGGGGTATAATAGTTACGACCAAGTAACTCAAGCAGGCAAGGCAGCGGTTGCCCG
TTTCTACGGTAGAGCAGCAGACAAAAGTTATTTCATCGGGTGTTCTGAAGGTGGGAGAGAAGGC
ATGATGCTTTCACAAAGATTCCCGTCTCACTACGACGGTATTGTAGCGGGCGCGCCAGGTTACC
AACTACCGAAAGCTGGTATATCCGGAGCTTGGACCACACAAAGCCTAGCTCCGGCGGCGGTCGG
CCTAGATGCTCAGGGAGTACCACTAATCAACAAGTCTTTTAGTGACGCGGATTTGCATCTACTT
AGTCAGGCTATCCTTGGCACATGCGATGCTTTAGATGGCTTGGCGGATGGAATCGTGGATAACT
ATCGTGCCTGTCAAGCTGCCTTTGACCCGGCCACGGCTGCGAATCCAGCTAACGGGCAGGCTCT
GCAGTGCGTGGGCGCCAAGACCGCCGATTGCTTGAGTCCCGTGCAAGTAACCGCCATCAAAAGA
GCAATGGCAGGCCCAGTAAATTCCGCAGGTACCCCGTTATATAATAGGTGGGCCTGGGACGCTG
GAATGAGCGGATTATCCGGCACAACGTATAACCAGGGGTGGCGTTCATGGTGGTTAGGTTCATT
TAATTCAAGCGCAAACAATGCTCAAAGAGTTAGTGGGTTTAGTGCCAGAAGCTGGCTTGTGGAT
TTCGCGACTCCTCCGGAACCAATGCCAATGACCCAGGTCGCCGCCAGGATGATGAAATTTGACT
TTGACATTGACCCTTTGAAGATCTGGGCAACCTCAGGCCAGTTTACCCAGAGCAGCATGGACTG
GCACGGTGCTACCTCAACTGACTTGGCTGCTTTCAGGGACAGAGGTGGCAAAATGATTCTTTAC
CATGGGATGAGTGACGCTGCCTTTAGCGCCTTAGATACTGCTGACTACTACGAGCGTCTGGGAG
CTGCAATGCCAGGGGCTGCCGGTTTTGCCCGTCTGTTTTTAGTTCCTGGTATGAACCATTGCTC
TGGCGGACCTGGAACTGATCGTTTCGATATGCTTACTCCACTTGTGGCCTGGGTGGAACGTGGC
GAGGCACCAGACCAGATCTCAGCCTGGAGCGGCACCCCAGGCTATTTCGGCGTGGCAGCAAGGA
CTAGGCCTTTGTGTCCCTACCCCCAAATCGCTAGATACAAGGGTTCTGGAGATATAAATACAGA
GGCAAACTTCGCTTGCGCCGCCCCTCCGggttctgctggttctgctgctggttctggtgaattt
ATGGTCAGTAAGGGTGAAGAATTATTCACTGGTGTTGTTCCAATCTTGGTTGAATTGGATGGTG
ATGTTAACGGTCACAAGTTTTCTGTTCGTGGTGAAGGTGAAGGTGATGCTACTAATGGTAAATT
GACCTTGAAGTTCATCTGTACCACAGGTAAATTGCCAGTTCCATGGCCAACTTTGGTTACTACT
TTGACTTATGGTGTCCAATGCTTCTCTAGATACCCAGATCATATGAAGCAACACGACTTTTTCA

AATCCGCTATGCCAGAAGGTTACGTTCAAGAAAGAACCATCTCTTTCAAGGATGACGGTACTTA
CAAAACTAGAGCCGAAGTTAAGTTCGAAGGTGATACCTTGGTTAACAGAATCGAATTGAAGGGT
ATCGACTTCAAAGAAGATGGTAACATCTTGGGTCATAAGTTGGAATACAACTTTAACTCCCACA
ACGTTTACATTACTGCCGATAAGCAAAAGAACGGTATCAAGGCTAACTTCAAGATCAGACACAA
CGTTGAAGATGGTAGTGTTCAATTGGCTGATCACTACCAACAAAACACTCCAATTGGTGATGGT
CCAGTTTTGTTGCCAGATAACCATTACTTGTCTACCCAATCTAAATTGTCTAAGGACCCAAACG
AAAAAAGAGATCACATGGTCTTGAAAGAACGTGTTACTGCTGCTGGTATTACTTTGGGTATGGA
CGAATTATACaaggaaaatttgtattttcaatctgaacaaaaattgatttctgaagaagatttg
[----------------------DISPLAY PARTNER ---------------------]
cagaagacgggagacactagcacacaactttaccaggcaaggtatttgacgctagcatgtgtcc
aattcagtgtcatttatgatttttttgtagtaggatataaatatatacagcgctccaaatagtgc
ggttgccccaaaaacaccacggaacctcatctgttctcgtactttgttgtgacaaagtagctca
ctgccttattatcacattttcattatgcaacgctttttcccgacgagagtaaatggcgaggata
cgttctctatggaggatggc

xxx: 5′ and 3′ insertion sites at CAN1
xxx: WTC846PR846 promoter
XXX: OST1 secretion signal
XXX: *Ideonella sakaiensis* MHETase
xxx: Linker
XXX: GFP
xxx: TEV protease site
xxx: myc tag
xxx: PRM9 terminator

Display partners sequences:
AGA2
CAGGAACTGACAACTATATGCGAGCAAATCCCCTCACCAACTTTAGAATCGACGCCGTACTCTT
TGTCAACGACTACTATTTTGGCCAACGGGAAGGCAATGCAAGGAGTTTTTGAATATTACAAATC
AGTAACGTTTGTCAGTAATTGCGGTTCTCACCCCTCAACAACTAGCAAAGGCAGCCCCATAAAC
ACACAGTATGTTTTTTtga

CCW12
GCAGCAAATGTAACAACAGCCACAGTATCACAAGAGTCTACGACACTTGTTACCATCACCTCCT
GCGAAGACCATGTCTGTTCTGAGACTGTGTCTCCTGCACTGGTCTCAACTGCCACTGTGACCGT
TGATGATGTCATTACCCAGTATACAACTTGGTGTCCCTTGACAACTGAAGCTCCAAAAAACGGC
ACTTCCACCGCAGCCCCAGTGACTAGTACAGAAGCACCAAAAAATACTACCTCAGCGGCTCCGA
CGCACTCGGTTACGAGTTACACAGGTGCCGCGGCAAAGGCACTCCCTGCTGCAGGTGCTTTATT
AGCTGGAGCTGCTGCTCTATTGTTGtga

CIS3
GACGTGATCTCACAGATTGGAGATGGACAAGTGCAAGCAACTTCTGCAGCTACCGCTCAAGCCA
CTGACTCTCAAGCTCAAGCTACTACTACAGCTACCCCAACTAGTTCCGAAAAGATTAGCTCCTC
GGCATCCAAGACTTCAACCAATGCAACCTCCTCTTCCTGTGCAACGCCAAGTTTGAAGGACAGT
TCTTGTAAAAATTCTGGTACGTTAGAACTCACGCTAAAGGATGGTGTATTGACTGATGCGAAAG
GGAGAATTGGATCGATTGTTGCCAATAGGCAATTTCAGTTTGATGGGCCGCCTCCACAAGCTGG
TGCTATATACGCCGCAGGTTGGTCAATTACAGAAGATGGCTACTTGGCTTTAGGCGATAGTGAC

GTTTTCTATCAGTGTCTATCAGGAAACTTTTACAACCTTTATGATCAAAATGTCGCTGAACAGT
GTAGCGCTATCCATCTGGAAGCTGTCAGCCTTGTTGATTGCtga

CWP2
GAGAGCGCCGCTGCAATATCTCAAATTACGGATGGACAGATCCAAGCGACCACAACAGCTACTA
CTGAAGCTACAACTACTGCCGCGCCATCTTCCACCGTTGAAACCGTATCCCCTAGTTCGACTGA
GACAATTTCTCAACAAACGGAAAACGGTGCTGCTAAAGCAGCTGTTGGGATGGGTGCTGGCGCA
CTTGCTGCAGCTGCAATGTTATTGtga

SED1
CAATTTTCCAACAGCACATCTGCTAGCTCTACAGACGTAACATCCAGCTCATCAATCTCGACAA
GTTCAGGTTCTGTTACAATAACGTCTTCTGAAGCGCCCGAATCCGATAACGGTACCAGTACAGC
AGCTCCAACTGAAACGAGCACGGAGGCGCCCACAACTGCCATTCCTACAAACGGTACTTCAACT
GAGGCACCAACTACCGCTATACCAACCAACGGCACCTCGACGGAAGCCCCTACTGATACAACAA
CTGAGGCACCTACGACCGCATTACCGACCAATGGTACTTCCACTGAGGCTCCTACAGACACCAC
GACAGAAGCTCCCACCACCGGATTGCCGACGAATGGAACAACTTCAGCCTTCCCCCCTACTACG
TCCTTGCCACCATCCAATACGACAACGACTCCGCCTTATAATCCATCTACAGATTATACGACAG
ACTATACAGTTGTAACTGAGTACACAACATATTGTCCTGAGCCAACCACATTCACTACTAACGG
CAAAACCTACACGGTCACTGAACCAACTACTCTAACAATCACTGATTGCCCGTGTACAATAGAA
AAACCTACTACCACGAGTACTACAGAATACACCGTAGTCACTGAATATACAACCTACTGTCCAG
AACCAACAACTTTTACCACCAATGGCAAGACCTATACTGTTACAGAACCAACAACCTTAACAAT
TACGGATTGCCCATGTACTATTGAGAAAAGTGAAGCTCCCGAATCCTCTGTTCCTGTCACAGAA
TCTAAAGGGACCACTACTAAGGAAACTGGTGTGACTACCAAGCAGACTACCGCCAATCCTTCTT
TGACTGTGTCGACTGTGGTGCCAGTTTCTTCTAGTGCATCAAGTCATTCAGTCGTTATTAATTC
AAACGGTGCTAATGTTGTCGTACCAGGAGCCTTAGGGCTGGCAGGAGTTGCTATGCTGTTTCTT
tga

TIP1
GATACTTCGGCTGCCGAAACTGCTGAGCTGCAGGCTATCATTGGGGACATTAATTCCCATTTAA
GTGATTATTTAGGTCTTGAAACTGGTAATTCTGGATTTCAAATCCCGTCAGACGTACTCAGTGT
GTATCAACAGGTGATGACTTACACAGATGATGCTTACACCACTTTGTTTTCTGAACTTGATTTC
GACGCAATAACCAAAACTATAGTCAAACTACCATGGTATACAACAAGATTATCATCAGAAATTG
CTGCTGCCTTGGCAAGCGTTTCGCCTGCTTCTTCCGAAGCAGCTAGTTCCTCGGAGGCTGCTTC
TAGTTCGAAGGCAGCGTCCTCTTCAGAAGCAACATCATCAGCTGCACCTTCATCTTCAGCCGCT
CCATCCTCTAGCGCTGCACCCTCTTCTAGTGCCGAATCATCTAGCAAGGCCGTTAGTTCCAGCG
TCGCCCCAACAACGAGTTCGGTGTCCACCTCTACGGTTGAGACCGCATCAAACGCAGGTCAAAG
GGTTAACGCAGGTGCAGCCAGCTTCGGCGCAGTTGTAGCTGGAGCGGCGGCGCTATTGTTAtga
